# An adverse event capture and management system for cancer studies

**DOI:** 10.1186/1471-2105-16-S13-S6

**Published:** 2015-09-25

**Authors:** Alex Lencioni, Laura Hutchins, Sandy Annis, Wanchi Chen, Emre Ermisoglu, Zhidan Feng, Karen Mack, Kacie Simpson, Cheryl Lane, Umit Topaloglu

**Affiliations:** 1 Division of Hematology and Oncology, University of Arkansas for Medical Sciences (UAMS), 4301 West Markham St., Little Rock, AR 72205, USA; 2 Winthrop P. Rockefeller Cancer Institute, University of Arkansas for Medical Sciences (UAMS), 4301 West Markham St., Little Rock, AR 72205, USA; 3 IT Research Systems, University of Arkansas for Medical Sciences (UAMS), 4301 West Markham St., Little Rock, AR 72205, USA; 4 Division of Biomedical Informatics, University of Arkansas for Medical Sciences (UAMS), 4301 West Markham St., Little Rock, AR 72205, USA

## Abstract

**Background:**

Comprehensive capture of Adverse Events (AEs) is crucial for monitoring for side effects of a therapy while assessing efficacy. For cancer studies, the National Cancer Institute has developed the Common Terminology Criteria for Adverse Events (CTCAE) as a required standard for recording attributes and grading AEs. The AE assessments should be part of the Electronic Health Record (EHR) system; yet, due to patient-centric EHR design and implementation, many EHR's don't provide straightforward functions to assess ongoing AEs to indicate a resolution or a grade change for clinical trials.

**Methods:**

At UAMS, we have implemented a standards-based Adverse Event Reporting System (AERS) that is integrated with the Epic EHR and other research systems to track new and existing AEs, including automated lab result grading in a regulatory compliant manner. Within a patient's chart, providers can launch AERS, which opens the patient's ongoing AEs as default and allows providers to assess (resolution/ongoing) existing AEs. In another tab, it allows providers to create a new AE. Also, we have separated symptoms from diagnoses in the CTCAE to minimize inaccurate designation of the clinical observations. Upon completion of assessments, a physician would submit the AEs to the EHR via a Health Level 7 (HL7) message and then to other systems utilizing a Representational State Transfer Web Service.

**Conclusions:**

AERS currently supports CTCAE version 3 and 4 with more than 65 cancer studies and 350 patients on those studies. This type of standard integrated into the EHR aids in research and data sharing in a compliant, efficient, and safe manner.

## Background

Data collection in cancer research studies is vital to understand the possible side effects of medication, the medication's efficacy, and overall safety in patients. Collecting AEs has become problematic with the use of both data management systems and EHR to record and monitor the adverse events in the study population. From the time the clinician documents the encounter with the study participant to the data entry into the database, there are several potential sites for AEs to be duplicated, incompletely documented, or transcribed incorrectly.

The Food and Drug Administration's Code of Federal Regulations Title 21 Section 312.64 requires investigators to immediately report any event to the sponsor regardless of the relationship to the drug [1]. When Belknap et al compared several nationwide cancer institutes looking at the quality of methods of adverse event reporting, they found zero of the 49 institutes "use a valid method for assessing causality," and 38 of the 49 are implicitly prompting global introspection by the investigator [2]. Only one-third of the forms provided the domain of terms recommended by the Food and Drug Administration for the assessment of quality. Belknap et al also noted that an integration of taxonomy system to CTCAE could help amend some of the issues within adverse event reporting. They recommended that because of the familiarity of the oncologist to use CTCAE [2]. The findings of Belknap reiterate the finding from a report in the Institute of Medicine on the national cancer clinical trials system. The report states that "the lack of a standard required data set leads to inconsistency in the data collected for cancer trials that can affect the quality of the study and limit cross-study comparisons." [3]

The study titled "Does error and adverse event reporting by physicians and nurses differ?" found that of all the events reported physicians only accounted for 1.1%, while reporting by nurses accounted for 45.3%. The study also found that physicians and nurses reported different types of events. Showing physicians more often reported events that "caused permanent harm, near death, or death of the patient" and nurses more often reported "events that caused no or temporary harm" [4]. Having a system where the adverse events are collected systematically and well-documented within the patient's chart can improve the recognition, evaluation, and monitoring of all adverse events by the entire patient care team [12,13].

A recent report compiled by the United States Department of Health and Human Services' Inspector General identifed very common issues on AE reporting citing the incomplete collection and information system related issues [7]. Although Epic has the highest EHR marketshare [6], our cancer clinics have determined that AE capture and reporting features of Epic are not as functional as AERS and are more difficult to track ongoing adverse events. Another alternative could be to use some other existing AE capture platforms; however, to our knowledge, there is no available AE capture system in the public domain. caBIG Adverse Event Reporting System (caAERS) [8] has been developed by the National Cancer Institute; however, it is a reporting system to generate necessary AE reports once an AE is captured and it is not a AE capturing platform. Due to those constraints, and since academic health centers have their own diverse IT infrastructructure, AERS was created to improve the efficacy of AE reporting at UAMS. The AERS system integrates with the EHR allowing the information to be documented within the patient encounter note in the legal medical records. AERS is interfaced to a research database as well and eliminates the need of the clinical coordinator to retype AEs, thus preventing transcription errors. Having the AE database and the EHR integrated, the AERS system prompts the clinician to address the ongoing AEs and note any changes. AERS will also allow clinicians to address new AEs. The clinician will be prompted to address all attributes regarding the ongoing or new AEs to ensure the AEs are documented completely. The AERS system has drastically improved AE reporting efficacy and has helped eliminate unnecessary data queries regarding data documentation by improving the data collection process. By decreasing the AE queries, the efficiency and accuracy of clinical trial management has improved.

## Methods

AERS was implemented as part of the Comprehensive Research Informatics Suite (CRIS) at UAMS and interfaced with the Epic EHR to provide easy integration to clinic workflows and properly document AEs in a patient's chart. As majority of academic health centers use Epic EHR [6], we believe, integration schema defined in this paper is easily adoptable. For non-Epic centers, the approach is modeled after the way an external transcription service would work, so it is possible for non-Epic EHR customers to take advantage of the AERS.

### CRIS

**Figure 1:**
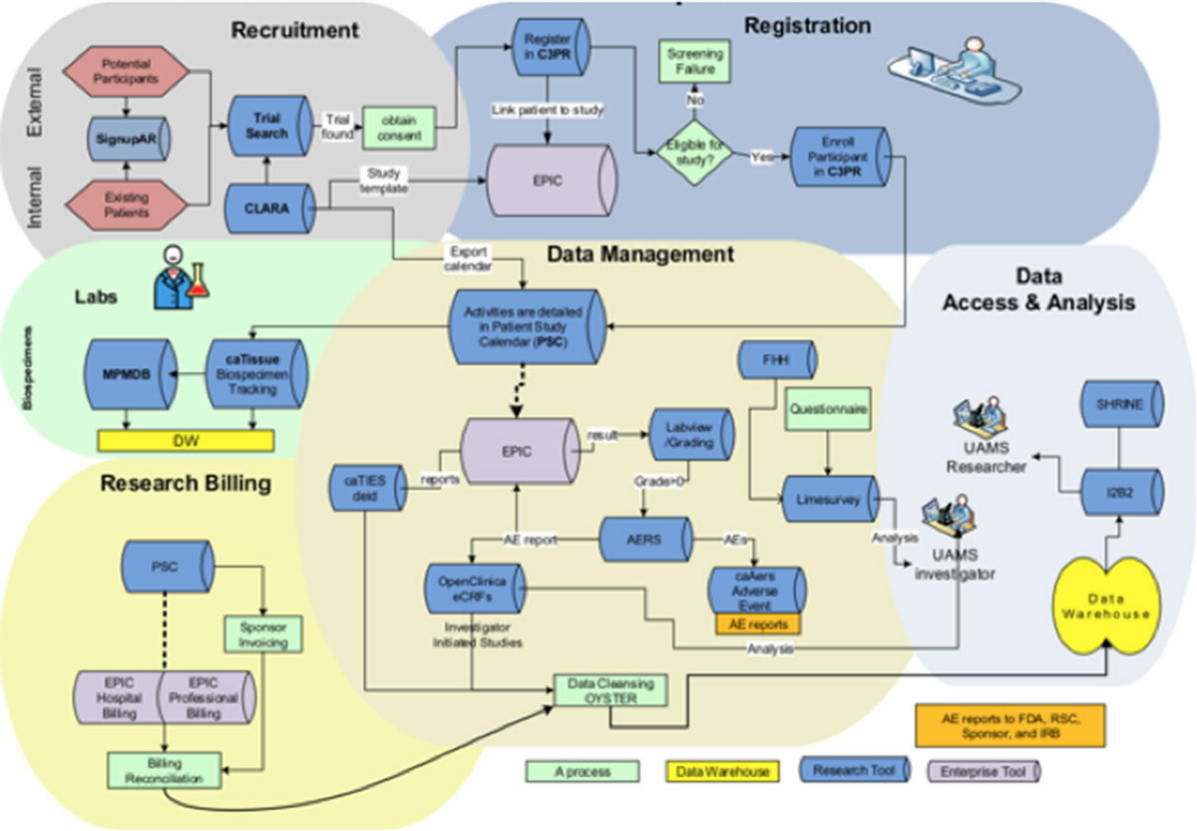
**Vision for Clinical and Translational Research Operational Workflow**.

A novel component for biomedical informatics coordination efforts at UAMS is the adoption of common research biomedical informatics systems; a goal toward which we have already made unparalleled progress with CRIS (Figure 1). Started as a Winthrop P. Rockefeller Cancer Institute initiative, CRIS consists of 27 systems that are integrated with an Enterprise Service Bus, with 23 tools coming from open-source projects, mainly the National Cancer Institute's cancer Biomedical Informatics Grid initiative and other public sources. CRIS is currently supporting 419 studies and 346 users from 43 groups on campus. As a single platform, CRIS has three major benefits: synergy, efficiency, and security. First, our experience has been that functionality developed for research in one domain (e.g., cancer research) nearly always benefits research in other domains (e.g., pediatrics, geriatrics, etc.). This has resulted in high levels of synergy in our work and reduced the cost of biomedical informatics support for research. Second, CRIS is a single platform that supports the entire translational spectrum--from specimen management applications for basic science research, to Phase 3 or 4 multi-site cancer trials. This enables researchers to move ideas, data, results, technology, etc. from bench to bedside more efficiently because they do not need to transition experiments, studies, trials, technology, and data to new software that might not be compatible with other platforms. Third, as a single platform, CRIS is considerably easier to secure because there is only one application for each task. All components of CRIS are web based, enabling sharing and integration of clinical research information for single and multi-site trials.

All applications are integrated into a portal that allows a single point of access with a registered UAMS username and password. All CRIS applications reside on a cluster server with failover capability behind the UAMS firewall, and thus have the benefit of high security, fire protection, and routine backup. CRIS modules are widely adopted across UAMS, and currently enhancing the suite to support their vanguard work in translating genomics and proteomics research into clinical practice.

### Symptoms and diagnosis

**Figure 2:**
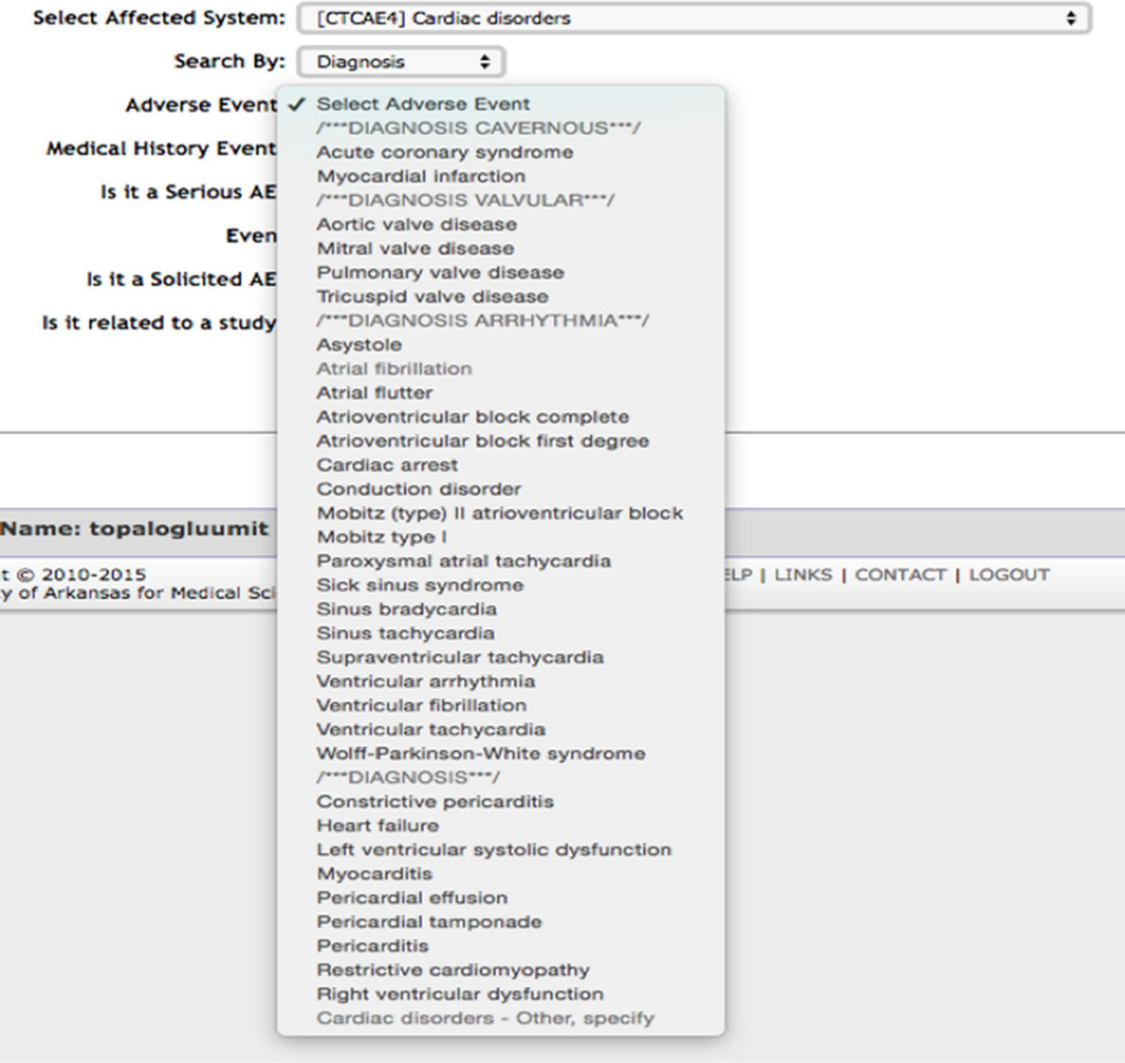
**Cardiac Disorders diagnosis list after re-categorization**.

AERS has incorporated a systematic way to separate the CTCAE into symptoms and diagnosis. When addressing new AEs, AERS will not allow the clinician to select an AE that is currently ongoing whether it was previously documented as a symptom or a diagnosis to eliminate duplicate reporting [10,11]. The adverse events listed in the CTCAE version 4 are listed by system, and the items are in alphabetical order. Since there are frequent synonyms used in clinical medicine, if the correct medical term does not come to mind it may be difficult to locate the item that applies to the adverse event. We rearranged the items in the same system heading by diagnosis (if the diagnosis had an ICD 9 code) or otherwise by symptom. In the diagnosis lists we further grouped the items under headings. For example, valvular heart disease diagnoses were under a subheading so titled and arrhythmias were named in a different subheading. This made the adverse events to be documented much easier to locate than an alphabetical list (Figure 2).

### AERS workflow

Current regulations require AEs to be captured and reported in a timely and as accurate as possible manner [1,7]. In order to increase the compliance, a user-friendly system is required that demands a bidirectional communication with the Epic EHR since Epic is the starting point for a patient encounter (i.e. visit).

In order to pull complete and accurate patient demographics of those registered into a study, we developed an interface to the caBIG Central Clinical Participant Registry (C3PR), which serves as the UAMS' research participant registry. C3PR also houses study personnel who have been approved to have access to participants' records and AERS authorizes the authenticated users and provides access to study personnel listed in C3PR only. CLinicAl Research Administrator (CLARA) is the UAMS' Institutional Review Board protocol management system that keeps study metadata (e.g. name, ID etc.) as well as the investigational drugs and devices if it is an interventional study. AERS pulls drug(s) or device(s) information from CLARA so that a clinican may properly associate any AE to the correct agent if AE is related.

There are two ways to create AEs in AERS: 1) Lab AEs that are auto generated based on the study participants' lab results (see section d;toxicity grading) and 2) office visits that are created by authorized users who assess any new or worsening symptom a patient may have due to study treatment (or pre-exisiting). In our workflow, a study nurse assesses the ongoing AEs and enters the new symptoms to make the system ready for the provider to finalize the assessment. There are small human interface reminders such as displaying a red frame for ongoing AEs that are not assessed during the visit yet to ensure all the ongoing AEs are designated as either ongoing or resolved.

Upon login to AERS, a physician will be provided list of awaiting assessments in a sidebar for easy access. The system sends email reminders to the physicians if AEs have not been finalized within 24 hours. The email reminders are very useful in case of lab-based AEs since lab results becomes available after an office visit and associated documentation is completed.

**Figure 3:**
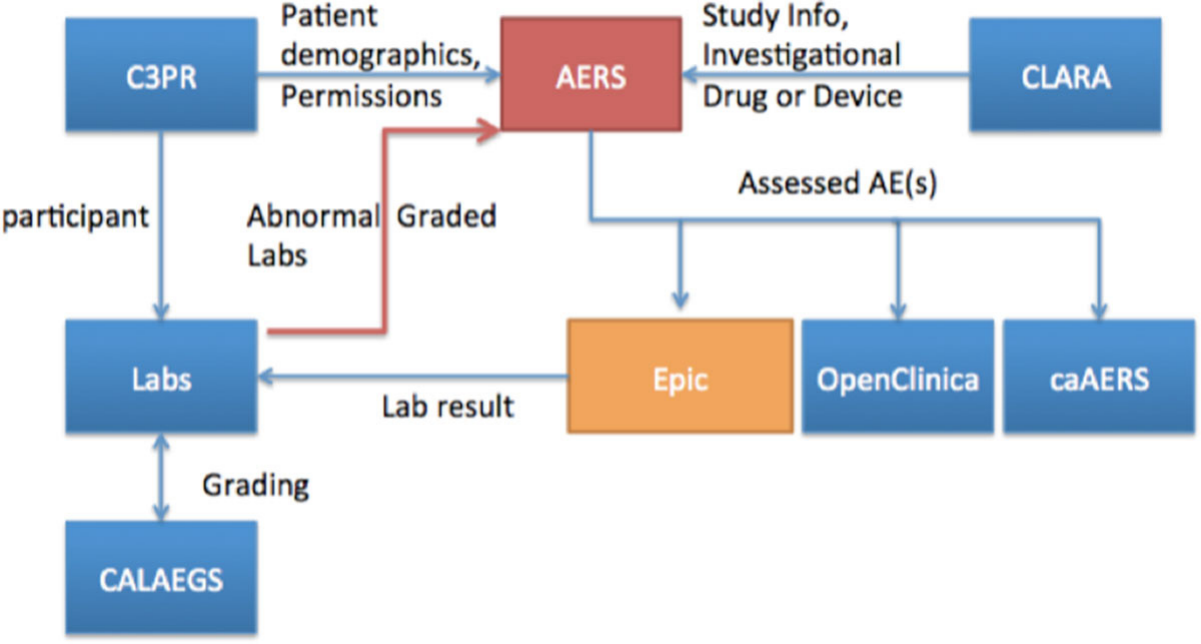
**AERS workflow**.

After a provider approves/completes the AE assesment, s/he is required to submit the assessment to the EHR, reporting tool, and Electronic Case Report Forms. Once the submit button is pressed, the system prompts the provider to select a Customer Serial Number, which is a unique identifier for each encounter in the Epic system to import assessment into the correct progress note. Customer Serial Numbers are stored in the CRIS' Patient Study Calendar, which was sent by the Epic's scheduling HL7 messages. AERS will gather all associated information from data sources of CRIS applications and generates documents by different assessment dates, AE types, and providers, then sends out in a defined format to Epic via HL7 and to the OpenClinica (CRIS' electronic case report form system) and caAERS system utilizing MirthConnect's web service and HL7 channels (Figure 3). MirthConnect is an open source interface engine that manages CRIS' all inter- and intra-system messages.

### Architecture of AERS

**Figure 4:**
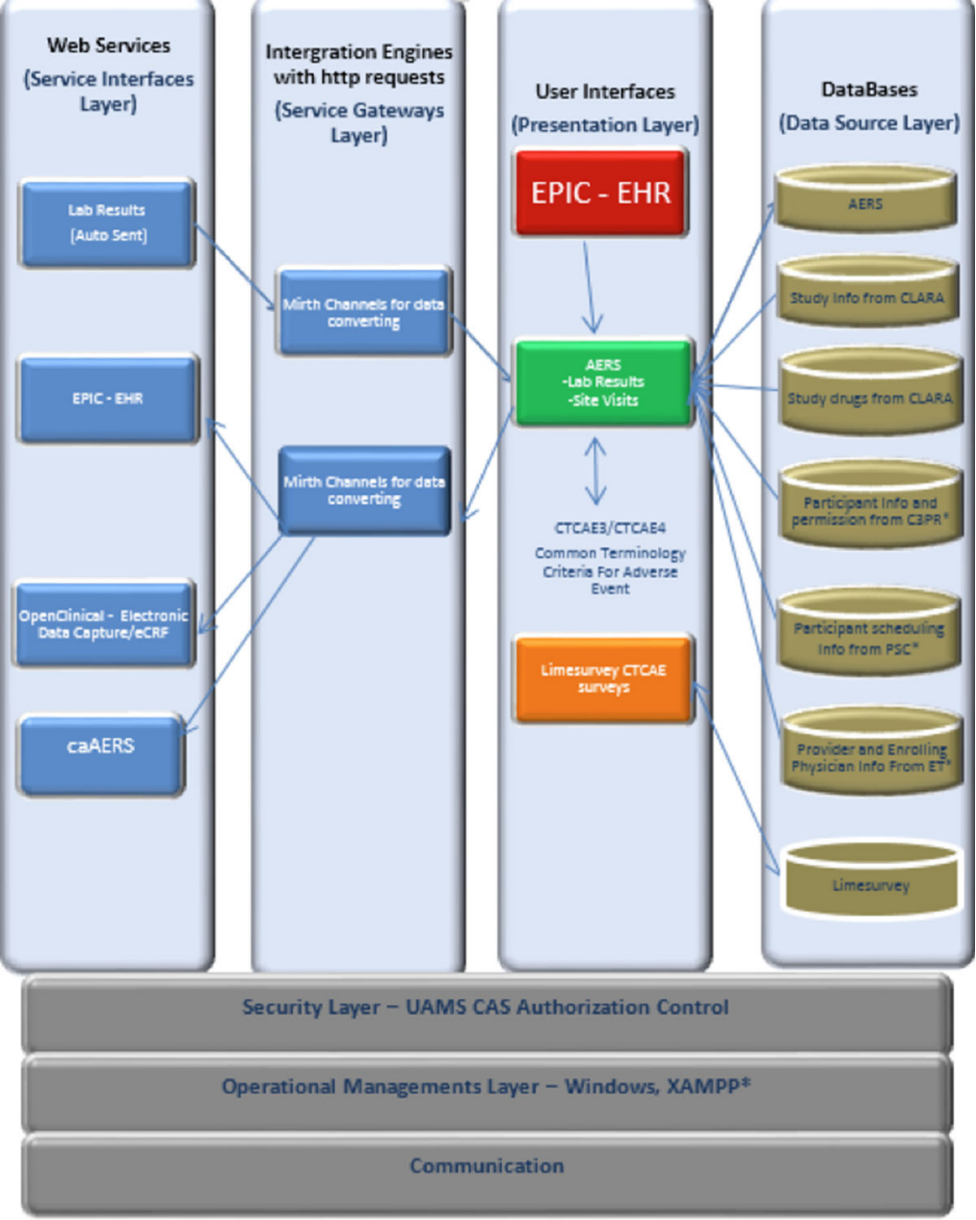
**AERS' architecture layers**.

AERS is designed to be a modular and scalable platform based on a multilayered architecture to accommodate a new version of the grading criteria with ease (Figure 4). At the operational management layer, it is running on a Windows 2012 server with an Apache + MySQL + PHP + Perl (XAMPP) environment. At the security layer, AERS verifies authentication against the Cental Authentication System that is interfaced to UAMS' Lightweight Directory Access Protocol. At the service interface layer, AERS has lab result, Epic EHR, OpenClinica, and caAERS web services. Authorized users can easily create new AEs that are categorized by affected systems, symptoms, and diagnoses. There are three user interfaces; 1) Epic, at which they launch AERS and stores the assessed AEs, 2) AERS where the assesment is completed, and 3) Limesurvey where the grading information for each AE is created. Since we use Limesurvey tool to create AE and associated grades, it is easily extendable to newer versions of CTCAE. Due to EHR changes, we had to program CTCAE v3, and it was completed with approximately 80 hours of analyst time. MySQL is the database for AERS as well as Limesurvey.

### Toxicity grading

**Figure 5:**
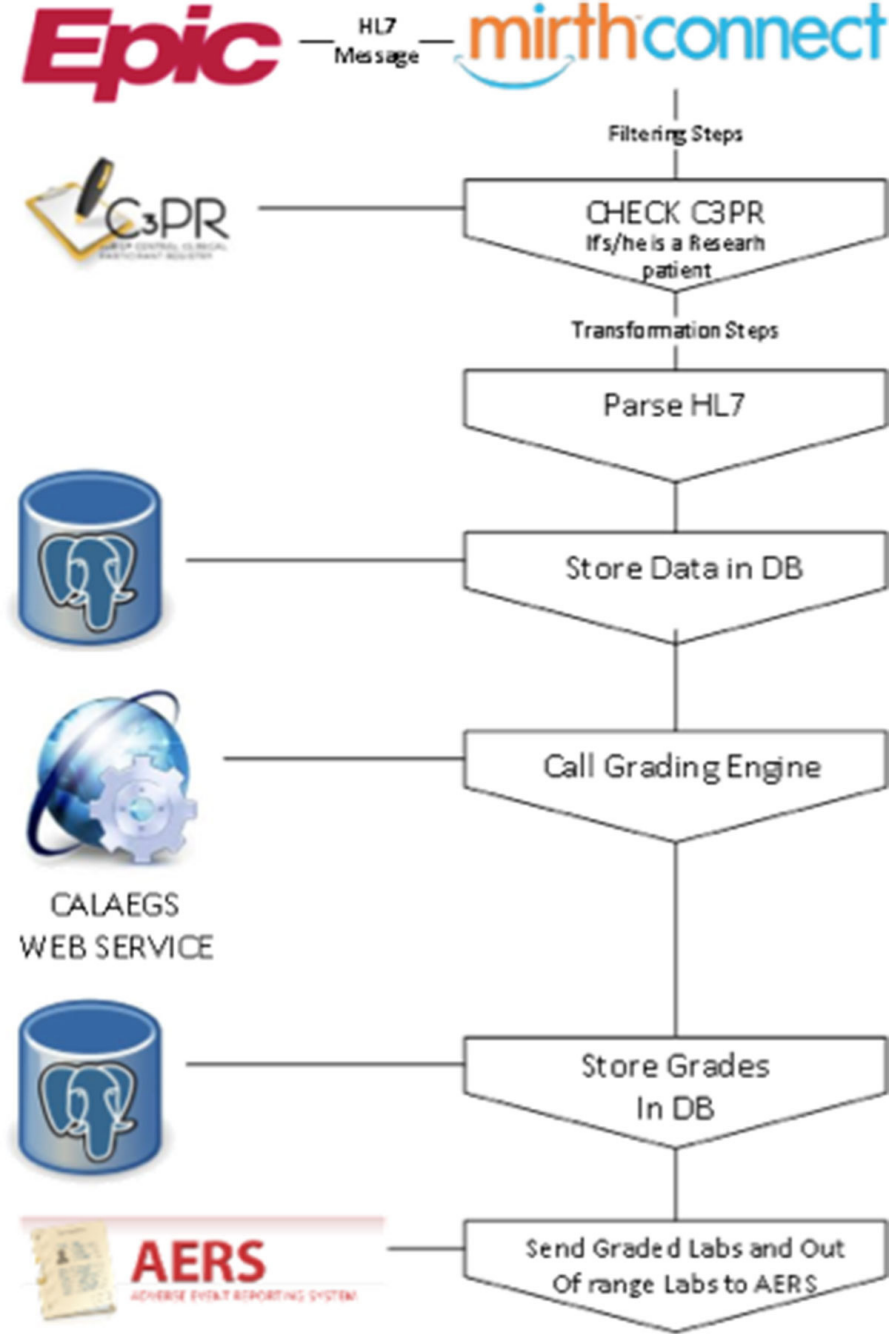
**Toxicity Grading Workflow**.

Given that many studies require lab results at all visits, properly tracking and grading all the labs is a cumbersome process and could be automated [5,9]. We have developed an automated system that grades all the numerically gradeable CTCAE labs and also record out-of-range values. It then sends both sets of information into the AERS so clinicians act upon them accordingly (Figure 5).

**Figure 6:**
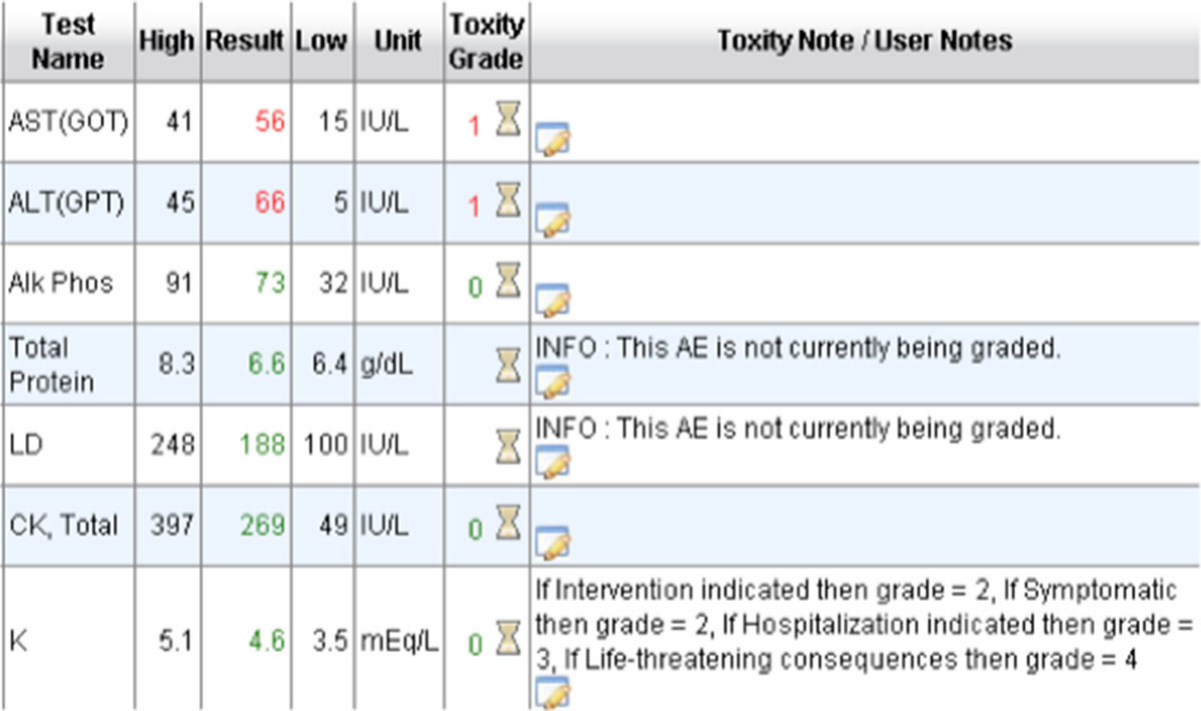
**Toxicity graded lab results view**.

A Minimal Lower Layer Protocol listener interface was created using MirthConnect interface engine. Epic EDI interface sends lab result messages to this interface as HL7 file (File format: ORU - R01). Each HL7 message goes through a different transformation and filtering process after it is accepted by MirthConnect interface engine. Upon receiving message, MirthConnect checks with CRIS' C3PR tool if the result belongs to one of the clinical research participants. If the result belongs to a subject, the MirthConnect starts parsing the HL7 message and stores the information in a database. In the next step, another transformation process sends this results to the Cancer Automated Lab-based Adverse Event Grading Service's (CALAEGS)[5] web service. This web service checks the result value, unit of measurement, lab type, and low - high values by using the predefined CTCAE rules and returns a grade as a response. Grades can be 0, 1, 2, 3, 4, or 5. Our system can process 28 different lab types out of 30 that are defined in CALAEGS. Some of the lab types have two defined Adverse Events. One for if the numeric value is less than lower limit of normal and one for if the numeric value is higher than upper limit of normal. For the potassium test (K), lower limit of normal is 3.5 mEq/L and upper limit of normal is 5.1 mEq/L. There are two defined adverse event for K. Those are hyperkalemia and hypokalemia. If result value is 3.3, the numeric value is less than lower limit of normal. For this case, defined CTCAE version 4.0 term is hypokalemia and the Medical Dictionary for Regulatory Activities' version 12.0 code is 10021018. On the other hand, if result value is 5.3, the numeric value is higher than upper limit of normal. For this second case, defined CTCAE v4.0 term is Hyperkalemia and the Medical Dictionary for Regulatory Activities' version 12.0 code is 10020647. Each adverse event is represented by the Medical Dictionary for Regulatory Activities' version 12.0 code. Responses coming from the CALAEGS web service is stored in a database. Another batch process checks this database and sends graded lab results and out-of-range lab results to the AERS web service for study association determination by the clinicians (Figure 5). There is also a lab and toxicity grading display interface for users to see grades in order to perform possible overwrites or grade (see Figure 6; K's numeric value is normal; however, additional information is needed for final grade, such as hospitalization.)

### Medical record integration and Epic interfaces

Epic supports Integrating the Healthcare Enterprise's Retrieve Process for Execution standard to enable providers to access research protocol with incoming messages from clinical research systems. Retrieve Process for Execution is one of a set of profiles that create interoperability between EHRs and clinical research systems. The study definition and the registration information are being sent to Epic via Retrieve Process for Execution from CRIS for all of the clinical research studies that are being conducted at UAMS' clinical enterprise. In addition, Epic has the bridge interfaces to allow incoming HL7 messages such as transcriptions. We utilize the research interface for AE documents, including lab-based AEs that are being generated by AERS and sent to as HL7 via the MirthConnect.

**Figure 7:**
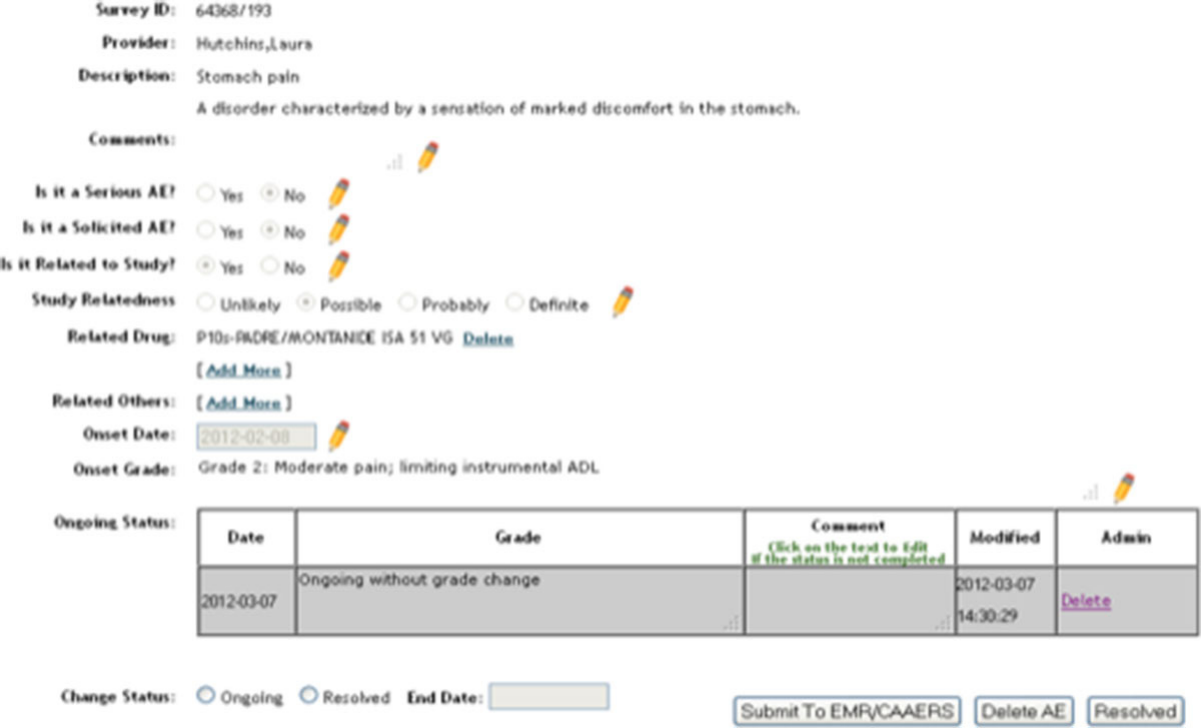
**AERS ongoing AE assessment view**.

**Figure 8:**
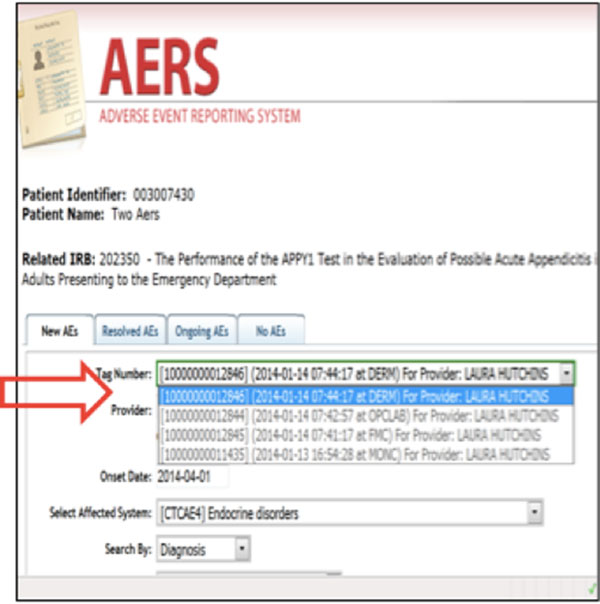
**AERS new AE entry**.

The workflow is as follows: in order to bring non-lab AE documentation into Epic from AERS, first a documentation note should be created with a visit, telephone, or documentation encounter in Epic. Then select the Research tab in the left navigational pane while in patient's encounter. Clicking the "Jump to AERS" link takes clinicians directly to the patient's ongoing assessment in AERS to ensure all the existing AEs are assessed (Figure 7). Upon completion, if there are additional symptoms (or diagnosis) to report, one should go the new AE tab within the AERS. The system disables all the existing AE terms, and there is a "tool-tip" displaying for each AE to give its description (Figure 8).

While adding a new AE, selecting the appropriate customer serial number (i.e. encounter) from the dropdown list is crucial to properly import incoming documents into Epic. This allows the system to route the AERS note to the appropriate encounter within Epic. Then the "complete AERS button and submit" buttons are selected.

**Figure 9:**
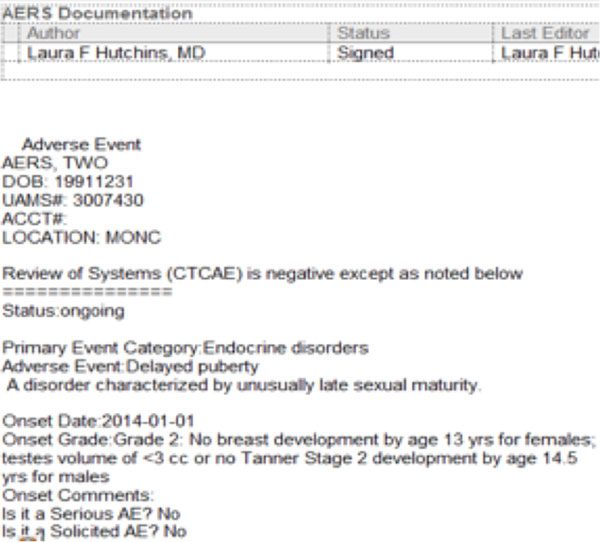
**Imported AE into the Epic progress note**.

AERS documentation appears in the provider's "in basket" folder in Epic, and must be signed by the provider. After signing, it is possible to type '.aers' within the encounter note in Epic to pull in all signed non-lab adverse event documentation for the encounter (Figure 9).

Lab-based AEs are filed as separate documents in Epic and automatically routed to providers' in basket for signature. They are then filed as Lab Based Adverse Event note types in Epic.

## Results and discussion

AERS has been directly implemented in more than 65 cancer studies and 350 patients on those studies within the UAMS oncology clinics. With the implementation of the AERS system, we have seen several improvements in the conduct of clinical trials. We have seen a decrease in the time study coordinators spend to complete case report forms related to adverse event reporting due to complete AE assessments being available in timely manner. The data is more organized, accurate, thorough, and easily monitored by sponsors. With the AERS system, electronic integration into the data collection database for local investigator-initiated trials allows direct upload of AE´s into the case report forms. This reduces the need for the study coordinators to transcribe data, and is the exact information electronically transcribed from the source document, which improves accuracy in data collection. The efficiencies and improvements that are attributed to the AERS are listed in Table 1.

**Table 1:** Improvements resulted by the AERS implementation.

#	Area of improvement	Estimated/actual improvement
1	Time spend for adverse event clarification between the clinical trial office staff and providers	Estimated 60% less inquiries to clinics for clarification

2	All ongoing AEs are being assessed every visit	The number of sponsor queries has decreased, which reduced the time needed to identify and complete missed and incomplete assessments.

3	Complete and accurate reporting of AEs	Staff spends less time to compile data from systems to complete needed attributes of an AE report. This allows more thorough and accurate reporting.

4	Timely reporting of AEs	As the complete and accurate AE report is easily available, the staff reports those in a timely manner as expected by the sponsors and regulatory bodies.

5	Complete reporting of the lab related AEs	Before AERS, the lab AE reporting was paper based with some potential missed lab-based AEs. Each lab AE had to be signed by the provider with proper association. After AERS, the research staff estimates that they are reporting 75% more lab-based AEs and providers just make the clinical significance determination and submit electronically.

6	Time saving and efficiency due to less number of queries.	We analyzed two studies and both of which have 10 subjects enrolled and the same investigational product, the time frame is from April 2008 to the present date. A) Study A that doesn't use AERS, had 106 queries out of which 73 were AE related (~69%). B) Study B that uses AERS, had 169 queries out of which 36 were AE related (27%)

The information is collected from the anecdotal and quantitative responses of the clinical trial office staff and the clinic staff

The reorganization of symptoms versus diagnoses and the ability to see ongoing AEs at the time of entering a possible new AE in AERS system decreases the chances of documenting the same AE twice under different medical synonyms. With the ability to place the AERS documentation in the encounter note, clinicians have an option to use it as the documentation for the review of symptoms section of the office visit. This eliminates descrepancies and the use of medical synonyms for the same event, which requires clarification in the note and queries to clarify the correct information. The combination of AERS with our workflow has addressed the discrepancies noted between nursing and physician assessments since the AEs collected by all healthcare team members are assessed at the same time with the same criteria, providing consistency in reporting and reducing global introspection with regard to assessing AEs. The feature that grades lab results and imports that into AERS for final assessment and attribution has saved much time and effort on the part of the clinicians, research coordinators and monitors. The elimination of transcription has reduced errors as well.

The design of the AERS system allows multiple sites to use the system for investigator initiated trials, thus providing the same consistent reporting, accurate data collection and improved efficacy of AE. With more accurate AE reporting we will be able to improve accuracy and reduce time, thereby ultimately leading to improved patient safety.

## Conclusions

The AERS system, which is integrated globally into cancer clinical trials, improves the efficacy and speed of AE reporting and promotes patient safety. The AERS system provides a platform to globally standardize AE reporting, ensuring consistent reporting, and will increase the efficiency of the clinical trial staff. This has been accomplished through the flexibility allowed by the software design with innovative integration of the open-source tools and the ability to import the data into the commercial EHR documentation. The flexible design may be enhanced in the future to allow patient-reported events, broadening the data collection further. We have used open-source tools and systems and the source code is available at https://github.com/vickiechen/AERS

## List of abbreviations used

AE: Adverse Event; AERS: Adverse Event Reporting System; CALAEGS: Cancer Automated Lab-based Adverse Event Grading Service; CLARA: CLinicAl Research Administrator; CRIS: Comprehensive Research Informatics Suite; CTCAE: Common Terminology Criteria for Adverse Events; EHR: Electronic Health Record; HL7: Health Level 7; ICD: International Classification of Diseases; K: Potassium; UAMS: University of Arkansas for Medical Sciences; XAMPP: Apache + MySQL + PHP + Perl.

## Competing interests

The authors declare that they have no competing interests.

## Authors' contributions

AL: Has written the introduction and background of the paper. LH: Is the subject matter expert and physician champion. Wrote sections for AE clasification and benefits. SA: used AERS for regulatory and reporting and provided some metrics for efficieny. WC: main AERS developer and provided architectural figures. EE: grading and interface developer. Wrote the grading section. ZF: technical lead and architect. KM: has done extensive testing and provided many usability and medical/regulatory feedback. KS: measured efficiency and tracked the metrics. Also used for monitoring purposes. CL: oversight and support for the AERS development. UT: Led the AERS implementation. Organized the paper and wrote implementation section of the paper.

## Declarations

Publication of this work was partly supported by the Winthrop P. Rockefeller Cancer Institute and Award UL1TR000039 from the National Center for Advancing Translational Sciences.

This article has been published as part of *BMC Bioinformatics *Volume 16 Supplement 13, 2015: Proceedings of the 12th Annual MCBIOS Conference. The full contents of the supplement are available online at http://www.biomedcentral.com/bmcbioinformatics/supplements/16/S13.
